# A Retrospective Multicenter Study of the Clinicopathological Characteristics and Prognosis of Young Adult Patients with Colorectal Cancer: Effects of Chemotherapy on Prognosis

**DOI:** 10.3390/jcm12113634

**Published:** 2023-05-23

**Authors:** Il Tae Son, Jae Hyun Kang, Byung Chun Kim, Jun Ho Park, Jong Wan Kim

**Affiliations:** 1 Department of Surgery, Hallym Sacred Heart Hospital, Hallym University College of Medicine, Anyang-si 445-907, Republic of Korea; 2 Department of Surgery, Dongtan Sacred Heart Hospital, Hallym University College of Medicine, 40, Sukwoo-Dong, Hwaseong-si 445-170, Republic of Korea; 3 Department of Surgery, Kangnam Sacred Heart Hospital, Hallym University College of Medicine, 948-1, 1, Shingil-ro, Yeongdeungpo-gu, Seoul 150-950, Republic of Korea; 4 Department of Surgery, Kangdong Sacred Heart Hospital, Hallym University College of Medicine, 445 Gil-1-dong, Gangdong-gu, Seoul 134-701, Republic of Korea

**Keywords:** colorectal cancer, young, pathological feature, prognosis

## Abstract

Background: The objective of this study was to evaluate clinicopathologic features of young patients with colorectal cancer (CRC) and to compare their prognosis with those of older patients Methods: We retrospectively reviewed the medical records of patients who underwent surgery for stage 0–III CRC at four university-affiliated hospitals between January 2011 and December 2020. The patients were divided into two groups, the young adult group (≤45 years) and the older group (>45 years). Results: Of 1992 patients, 93 (4.6%) were young adults and 1899 (95.3%) were older patients. Young patients showed more symptoms (*p* = 0.014) and more poorly or undifferentiated adenocarcinoma (*p* = 0.047) than older patients. The young adult patients were more likely to receive adjuvant chemotherapy (*p* < 0.001) and multidrug agents (*p* = 0.029), and less likely to cease chemotherapy (*p* = 0.037). The five-year RFS (recurrence-free survival) rate was better in the young adults than in the older patients (*p* = 0.009). In the multivariable analysis, young age was a significant prognostic factor for better RFS (*p* = 0.015). Conclusions: Young patients with CRC had more symptoms, aggressive histological features than older patients. They received more multidrug agents and discontinued chemotherapy less often, resulting in better prognosis.

## 1. Introduction

Colorectal cancer (CRC) remains the second leading cause of cancer deaths, with an estimated 940,000 deaths per year worldwide [1]. Several reports have described a recent reduction in the incidence and mortality rate of CRC that could be partly attributed to the adoption of regular screening programs for individuals aged ≥ 50 years [2,3]; however, the incidence of early onset CRC, among individuals aged < 50 years, has steadily increased worldwide [4,5,6]. Although several studies have agreed on the need for earlier screening, these studies differed in terms of when to start screening and the screening modality [7,8,9]. A retrospective analysis of the Surveillance, Epidemiology and End Results (SEER) database recommended colonoscopic screening from 40 years of age [7]. Schellerer et al. recommended rigid rectoscopy from 40 years of age, whereas sigmoidoscopy with fecal occult blood tests or colonoscopy may be performed in selected patients only [8].

Many studies have reported various histopathological and molecular features of young patients with CRC, including higher proportions of more-advanced, poorly differentiated, and mucinous cancers and fewer mismatch repair genes than older patients [10,11,12,13,14,15,16,17,18,19,20]. Although the clinical guidelines have not yet developed specific recommendations for young CRC patients, the National Comprehensive Cancer Network guidelines recommend more-extensive colectomy in CRC patients aged <50 years [21]. In addition, the National Bowel Cancer Audit noted that younger patients are more likely to undergo chemotherapy and long-course radiotherapy [22]

However, the oncological outcomes of young patients with CRC remain controversial. Several studies have reported that younger patients have a worse prognosis due to the advanced stage, aggressive features, and delayed diagnosis of their disease [12,17,19,20,23], whereas others have shown that the prognosis of young patients is similar to that of older patients [18,24,25,26]. Some studies have even reported that younger patients have a better prognosis than older patients [10,14,16]. Quah et al. suggested that in younger patients a greater number of retrieved lymph nodes and higher rates of chemotherapy contribute to favorable prognosis [27].

A possible reason for these differences is that the previous studies used different cut-off values to define the “young” age group, including 30, 35, 40, and 45 years, which could lead to different results and prognoses [10,11,12,13,15,16,17,18,20,24,25]. Discrepancies may also be caused by other factors, including different tumor stages at inclusion (stage I–III or I–IV, or only IV), the various countries examined (USA, Europe, Republic of Korea, Japan, and China), and the inclusion of hereditary CRC (familial adenomatous polyposis (FAP), hereditary nonpolyposis colorectal cancer (HNPCC)).

The objective of this study was to analyze the clinical and pathological characteristics of young adult patients with CRC. We also compared the survival outcomes between young and older patients to identify the risk factors for CRC prognosis.

## 2. Materials and Methods

### 2.1. Study Population

We retrospectively reviewed the medical records of patients who had undergone curative resection for stage 0–III CRC at four hospitals affiliated to Hallym University (Kangdong Sacred Heart Hospital, Hallym Sacred Heart Hospital, Kangnam Sacred Heart Hospital, and Dongtan Sacred Heart Hospital) between January 2011 and December 2020.

The patients were divided into two groups on the basis of a cut-off age of 45 years, i.e., a young adult group and an older group. Patients with incomplete data, FAP, HNPCC, or stage IV cancer were excluded from the study. Patients who underwent palliative surgery, including stoma construction or bypass, and patients undergoing local resection were also excluded.

### 2.2. Data Collection

The patient characteristics, perioperative variables, and pathologic results were retrieved from their medical records. The patient characteristics included age, gender, American Society of Anesthesiologists (ASA) score, body mass index (BMI), carcinoembryonic antigen (CEA), location of the primary tumor, symptoms, and the presence of obstruction or perforation. Tumor location was defined as the right-sided colon (from cecum, to transverse colon), left-sided colon (from splenic flexure, to rectosigmoid colon), or rectum. Symptoms included abdominal pain, dyspepsia, hematochezia or melena, bowel habit changes, and bodyweight loss. As described in our previous study, the colonic obstruction diagnoses were based on clinical symptoms and radiological evidence or endoscopic finding [28]. We defined colonic perforation mainly on the basis of radiological images, such as free air on plain X-ray or computed tomography (CT), together with symptoms and signs indicating peritonitis, including abdominal pain, fever, and leukocytosis.

The perioperative variables included operation time, emergency surgery, minimally invasive surgery (MIS), diversion, post-operative hospital stay, complications, mortality within 30 days, and chemotherapy. For chemotherapy, we evaluated the administration or discontinuation of chemotherapy, and the chemotherapy regimens were either a 5-fluorouracil-based single agent and oxaliplatin or irinotecan-hydrochloride-based multidrug agents.

The pathologic results included the histological type of the cancer, lymphovascular invasion (LVI), perineural invasion (PNI), the number of harvested lymph nodes, and the TNM stage. The tumor stage was defined according to the eighth edition of the American Joint Committee on Cancer TNM staging system [29].

### 2.3. Follow-Up

After discharge, the patients were followed-up via physical and laboratory examinations, including CEA and cancer antigen (CA) 19-9 tests, every 3–6 months for the first 2 years and every 6 months thereafter until 5 years had elapsed. Chest and abdominopelvic CT were performed every 6 months until 5 years had elapsed. Colonoscopy was performed at 1 year and then biennially during the follow-up period.

### 2.4. Outcome Measures

The long-term oncological outcomes comprised overall survival (OS) and recurrence-free survival (RFS). OS was defined as the time between cancer-related surgery and death from any cause or the date of the last follow-up. RFS was defined as the time between cancer-related surgery and disease recurrence or death from any cause.

The primary endpoint was to evaluate the clinical and pathological features of young adult patients. The secondary endpoints were to compare the 5-year RFS rate between the young adult patients and older patients and to identify factors affecting their prognosis.

### 2.5. Statistical Analysis

All statistical analyses were performed using SPSS version 26.0 (SPSS, Chicago, IL, USA). Categorical variables are presented as numbers and percentages of patients and were analyzed using Fisher’s exact test or the χ^2^ test, as appropriate. Continuous variables are presented as means and standard deviations and were analyzed using Student’s *t* test or the Mann–Whitney *U* test, as appropriate. OS and RFS were analyzed using the Kaplan–Meier method and compared using the log-rank test. Cox’s proportional hazards regression models were used to identify the prognostic factors for OS and RFS. The factors tested included age (≤45 years), gender (men), ASA class (≥3), obstruction, tumor location (rectal cancer), T stage (T4), lymph node metastasis, PNI, LVI, histological grade (poor/undifferentiated), and chemotherapy regimen. A value of *p* < 0.05 was considered statistically significant.

## 3. Results

During the study period of approximately 10 years, a total of 2502 patients with CRC underwent surgery in the hospitals and were included in the study. We excluded 246 patients with stage IV disease, 56 patients with incomplete data, 12 patients with FAP or HNPCC, 111 patients who underwent bypass surgery or stoma construction for palliation, and 85 patients who underwent trans-anal resection. After excluding these 510 patients, 1992 patients were eligible for the study. In total, 93 (4.6%) were young adults and 1899 (95.3%) were older patients.

### 3.1. Patients’ Charateristics

The patients’ characteristics are summarized in Table 1. The mean ages of the patients in the young adult and older groups were 38.8 and 67.7 years, respectively (*p* < 0.001). There were no differences in gender, BMI, CEA, family history of cancer, and family history of colorectal cancer between the two groups. The proportion of patients with ASA class 3 or 4 was much higher in the older patient group than in the young adult group (*p* < 0.001). The proportion of patients presenting with symptoms was higher in the young adult group than in the older group (68.8% vs. 55.9%, *p* = 0.014), whereas the proportion of patients diagnosed with a regular screening program tended to be higher in the older group (41.3% vs. 32.3%, *p* = 0.084). Among the symptoms considered, abdominal pain was significantly more frequent in the young adult group than in the older group (44.1% vs. 22.1%, *p* < 0.001), as was bodyweight change (7.5% vs. 3.0%, *p* = 0.016). Obstruction (24.7% vs. 14.2%, *p* = 0.010) and perforation (5.4% vs. 1.7%, *p* = 0.027) were also more frequent in the young adult group than in the older group.

### 3.2. Perioperative Outcomes

The perioperative outcomes are listed in Table 2. Although emergency surgery was performed more frequently in the young adult group than in the older group (16.1% vs. 8.5%, *p* = 0.011), there were no significant differences between the two groups in terms of MIS, stoma formation, post-operative hospital stay, or 30-day mortality. The complication rate and proportion of patients with ≥2 complications were similar in both groups. Compared with the older patients, the young adult patients were more likely to receive adjuvant chemotherapy (62.4% vs. 43.5%, *p* = 0.001) and multidrug agents (74.1% vs. 59.7%, *p* = 0.029); however, the proportion of patients who discontinued chemotherapy was higher in the older group than in the young adult group (20.0% vs. 8.8%, *p* = 0.037).

### 3.3. Pathologic Outcomes

The frequency of LVI, the number of harvested lymph nodes, and the T, N, and TNM stages were similar in both groups (Table 3). The proportions of patients with poorly differentiated or undifferentiated tumors (*p* = 0.010) and PNI (*p* = 0.047) were higher in the young adult group than in the older group.

### 3.4. Prognosis

**Figure 1:**
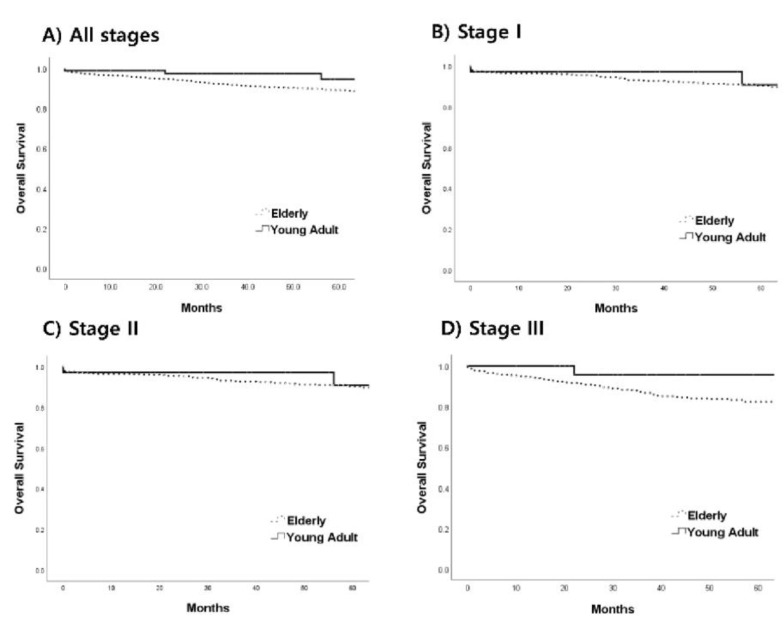
Comparison of five-year overall survival between the young adult and elderly patients for all stages ((**A**): 94.6% vs. 89.1%, *p* = 0.067), stage I ((**B**): 100% vs. 94.6%, *p* = 0.327), stage II ((**C**): 90.7% vs. 90.4%, *p* = 0.624), and stage III ((**D**): 95.7% vs. 82.4%, *p* = 0.102) colorectal cancer.

The mean duration of follow-up was 47.8 months (range, 2–108 months). The five-year OS rate in the young adult group tended to be better than that in the older group (94.6% vs. 89.1%, *p* = 0.067; Figure 1A). When patients with CRC were analyzed separately by stage (i.e., I–III), the five-year OS did not differ significantly between the two groups among those with stage I (young adult group vs. older group: 100% vs. 94.6%, *p* = 0.327; Figure 1B), stage II (90.7% vs. 90.4%, *p* = 0.624; Figure 1C), or stage III (95.7% vs. 82.4%, *p* = 0.102; Figure 1D) CRC.

**Figure 2:**
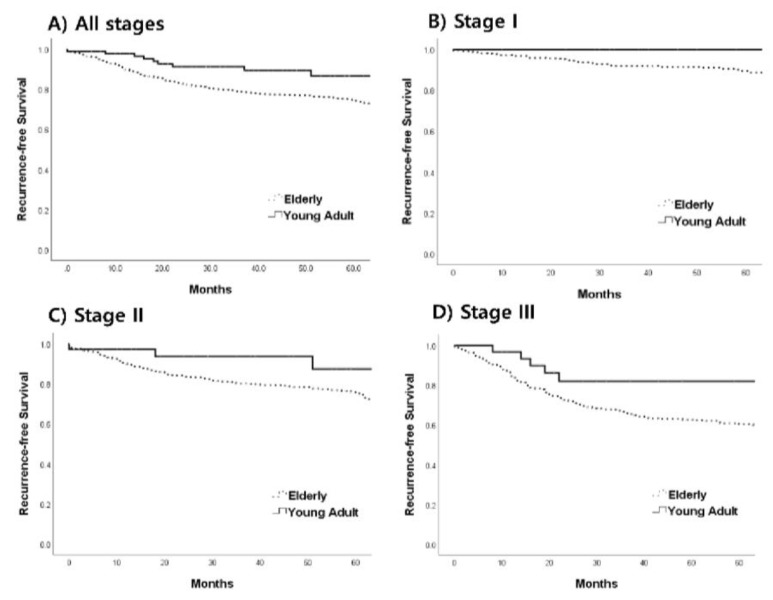
Comparison of five-year recurrence-free survival between the young adult and elderly patients for all stages ((**A**): 86.7% vs. 74.2%, *p* = 0.009), stage I ((**B**): 100% vs. 89.5%, *p* = 0.174, stage II ((**C**): 87.4% vs. 75.2%, *p* = 0.071), and stage III ((**D**): 82.0% vs. 60.3%, *p* = 0.048) colorectal cancer.

The five-year RFS rate was better in the young adult group than in the older group (86.7% vs. 74.2%, *p* = 0.009; Figure 2A). A subgroup analysis of patients by CRC stage showed that the young adult group had similar five-year RFS in stage I (89.5% vs. 100%, *p* = 0.174; Figure 2B), tended to have a better RFS in stage II (75.2% vs. 87.4%, *p* = 0.071; Figure 2C), and had a better RFS in stage III (60.3% vs. 82.0%, *p* = 0.048; Figure 2D) compared with the older group.

### 3.5. Factors Affecting Prognosis

We evaluated the risk factors for OS and RFS, and our results are shown in Table 4 and Table 5, respectively. Univariate analysis showed that ASA class ≥ 3 (*p* < 0.001), obstruction (*p* < 0.001), T4 (*p* = 0.001), the presence of lymph-node metastasis (*p* < 0.001), LVI (*p* < 0.001), and poor/undifferentiation (*p* = 0.015) were associated with worse OS. In the multivariable analysis, ASA class ≥ 3 (*p* = 0.001), obstruction (*p* = 0.001), rectal cancer (*p* = 0.026), T4 (*p* = 0.010), presence of lymph node metastasis (*p* < 0.001), and LVI (*p* = 0.003) were independently associated with worse OS, whereas chemotherapy (*p* < 0.001) was associated with better OS. Age (≤45 years) was not significantly associated with OS (*p* = 0.162; Table 4).

Regarding RFS, we found that ASA class ≥ 3 (*p* < 0.001), obstruction (*p* < 0.001), rectal cancer (*p* < 0.001), T4 (*p* < 0.001), presence of lymph node metastasis (*p* < 0.001), PNI (*p* < 0.001), LVI (*p* < 0.001), poor/undifferentiation (*p* = 0.002), and chemotherapy (*p* < 0.001) were independently associated with worse RFS in the univariate analysis. In the multivariable analysis, ASA class ≥ 3 (*p* < 0.001), obstruction (*p* < 0.001), rectal cancer (*p* < 0.001), T4 (*p* < 0.010), presence of lymph node metastasis (*p* < 0.001), and LVI (*p* < 0.001) were independently associated with worse RFS, and chemotherapy (*p* = 0.002) was associated with better RFS. Age (≤45 years) was a significant prognostic factor for better RFS (*p* = 0.015; Table 5).

## 4. Discussion

In the present study, we analyzed 93 young adult patients aged ≤45 years with CRC, who showed more symptoms (abdominal pain, obstruction, and bodyweight change) and more aggressive histological features (poorly or undifferentiated adenocarcinoma and positive PNI) than older patients. The young adult patients more frequently received adjuvant chemotherapy than the older patients, with a higher proportion of multidrug agents and fewer patients discontinuing chemotherapy, which resulted in a better RFS compared with the older patients.

A definition of “young” patients with CRC has not been established. Previous studies have used different cut-off values for age, including 30, 35, 40, 45, and 50 years [10,11,12,13,14,15,16,17,18,20,23,24,27,30,31]. If the cut-off age is lowered, the proportion of young patients in the total CRC population is reduced, which may lead to discrepancies among studies. The proportion of young patients in the total CRC population also differs according to region, e.g., in Western countries [10,11,12,14,15,16,18,23,27,31] and Asia [12,17,20,24]. Fu et al. divided all patients into six groups according to age and investigated a suitable cut-off age to define young adult patients with CRC. That study ultimately deemed 35 years a suitable cut-off age for “young” patients with CRC [17].

Previous studies have reported aggressive histological features in young patients with CRC, including poor differentiation and mucinous or signet-ring-cell-type tumors [12,13,14,15,16,18,20,26,31]. Unlike well and moderately differentiated tumors, these histological features are associated with advanced tumor stages [32]. In several studies, the frequency of LVI, a risk factor for poor prognosis of CRC patients, was significantly higher in young patients than in older patients [10,13]. In the present study, a higher proportion of young adult patients with CRC had poorly differentiated or undifferentiated carcinomas compared with the older patients (*p* = 0.010), and the PNI rate was higher in the young adult patients (*p* = 0.047).

Older patients are more likely to be diagnosed with CRC during regular screening, whereas young patients with CRC are more likely to present with symptoms. A delay in the diagnosis of cancer until symptom onset may result in the detection of more-advanced disease than if the cancer is diagnosed during regular screening [20,33,34]. Kim et al. reported that the interval between symptom onset and diagnosis was longer in young patients than in middle-aged patients (52.9 vs. 33.2 days), and the proportion of patients with a delayed diagnosis (≥3 months) was also higher in the young group than in the middle-aged group (14.9% vs. 7.9%, *p* < 0.01) [20]. In the present study, the proportion of diagnoses made during regular screening tended to be higher in the older group than in the young group (*p* = 0.084), and the frequency of symptoms, including abdominal pain and bodyweight change, was higher in the young adult group than in the older group (*p* = 0.014). The present study also showed higher rates of obstruction (*p* = 0.010) and perforation (*p* = 0.027) in the young adult group, which could be associated with the high rate of emergency surgery (*p* = 0.011). Accordingly, most previous studies have reported that because the diagnosis of cancer was often delayed, the young patients were diagnosed at a more advanced stage than older patients [10,13,14,16,17,18,20,25].

In the present study, young adult patients were more likely to receive adjuvant chemotherapy than the older patients (*p* = 0.001), which is consistent with previous studies [10,12,13,24]. Steele et al. divided their study subjects into four age groups (<40, 40–49, 50–79, and ≥80 years) and evaluated the impact of age on the treatments and outcomes. In patients with either stage II or III colon cancer, chemotherapy use decreased with increasing age (stage II: from 69.2% to 5.6%, *p* < 0.001; stage III: from 82.4% to 25.6%, *p* < 0.001) [15]. These results might be explained by the fact that young patients are more likely to tolerate chemotherapy-induced toxicity and have a better performances status than older patients [35,36]. In the present study, the rate of discontinuation of chemotherapy was higher in older patients than in the young adult patients (*p* = 0.037), and the young adult patients received a higher proportion of multidrug regimens than the older patients (*p* = 0.029).

The prognosis of young patients with CRC remains controversial. Several studies have reported better prognoses in young patients with CRC than in older patients [10,14,16]. In an analysis of 69,835 patients in the SEER database, the five-year CRC-specific survival of young patients was significantly better than that of older patients (78.6% vs. 75.3%, *p* < 0.001), although the young patients presented with unfavorable pathological features, including higher frequencies of poorly differentiated or undifferentiated tumors, mucinous or signet-ring-cell cancers, and stage III cancer [16]. A recent population-based study reported that although younger patients (≤40 years) were more likely to have LVI, T3/T4 tumors, and stage III cancer than older patients (>60 years), they had better OS (80% vs. 59%, *p* < 0.001) and cancer-specific survival (82% vs. 68%, *p* < 0.001). The authors called this result “paradoxical” and tentatively attributed it to a lower incidence of comorbidity, fewer postoperative complications, and improved tolerance of adjuvant chemotherapy in young patients [10]. Consistent with that study, the present study indicates that young adult patients tended to have a better five-year OS (*p* = 0.067) and five-year RFS (*p* = 0.009) than older patients. Despite the similar tumor stages in the two groups, the improved survival outcomes in the young patients might be attributable not only to the higher rate of chemotherapy but also to the active chemotherapy received by them, including a higher rate of multidrug use and a lower rate of chemotherapy discontinuation. Our multivariable analysis showed that chemotherapy was associated with better OS (hazard ratio (HR) = 0.344, *p* < 0.001) and RFS (HR = 0.696, *p* = 0.002).

There were several limitations to this study. First, it was a retrospective study, so several variables may have been inadequately recorded. Second, compared with several population-based studies, the relatively small number of patients in this study may have limited our ability to draw definitive conclusions; however, we extracted more-detailed patient data, including clinical variables (types of symptoms, family history), pathology results (presence of LVI or PNI), and treatment outcomes (length of hospital stay, complications, chemotherapy regimen, and administration/discontinuation) than other studies. Finally, the cut-off value of 45 years was selected arbitrarily for the young patient group, although several previous studies have used the same cut-off value to define young patients [11,20,23]; however, the percentage of young patients (4.6%) was similar to that in population-based studies, in which it ranged from 4.3% to 6.2% [15,16,18]. In a future study, the lack of a definition of young patients with CRC will be addressed, and young patients with stage IV CRC will be analyzed. Despite these limitations, this study is one of the few studies to compare the clinical and pathological features of young adult CRC patients (≤45 years) with those of older patients. In particular, this study described the effect of continuous and multidrug-based chemotherapy on the prognosis of CRC.

## 5. Conclusions

In this study, the analysis of 93 young adult patients (aged ≤ 45 years) with CRC showed that young patients had more symptoms, poorly or undifferentiated adenocarcinomas, and a higher rate of PNI than older patients. The young patients received more multidrug agents and discontinued chemotherapy less often, resulting in better RFS than that of older patients.

## Figures and Tables

**Table 1:** Patient characteristics according to age.

	Young Adult Group (n = 93)	Older Group (n = 1899)	*p*
Age	38.8 (±6.1)	67.7 (±10.6)	<0.001
Gender			0.192
Men	48 (51.6)	1110 (58.5)	
Women	45 (48.4)	789 (41.5)	
ASA			<0.001
I	43 (46.2)	196 (10.3)	<0.001
II	43 (46.2)	1102 (58.0)	
III/IV	7 (7.5)	601 (31.6)	
BMI (kg/m^2^)	23.2 (±3.2)	23.5 (±3.4)	0.391
CEA (ng/mL)	8.3(±12.4)	11.0 (±43.7)	0.443
Location			0.444
Right colon	30 (32.3)	576 (30.4)	
Left colon	36 (38.7)	683 (36.0)	
Rectum	27 (29.0)	638 (33.6)	
Symptom	64 (68.8)	1062 (55.9)	0.014
Abdominal pain	41 (44.1)	420 (22.1)	<0.001
Hematochezia/melena	13 (14.0)	392 (20.6)	0.119
Bowel habit change	12 (12.9)	225 (11.8)	0.759
Body weight change	7 (7.5)	57 (3.0)	0.016
Dyspepsia	3 (3.2)	51 (2.7)	0.738
Tenesmus	2 (2.2)	16 (0.8)	0.204
obstruction	23 (24.7)	270 (14.2)	0.010
Perforation	5 (5.4)	32 (1.7)	0.027
Regular screening	30 (32.3)	784 (41.3)	0.084
Family history of cancer	15 (16.1)	238 (12.6)	0.314
Family history of CRC	3 (3.2)	4.2 (2.2)	0.523

Data are presented as the number of patients (%) or mean (±standard deviation) unless otherwise stated. n, number; ASA, American Society of Anesthesiologists; CRC, colorectal cancer.

**Table 2:** Perioperative outcome according to age.

	Young Adult Group (n = 93)	Older Group (n = 1899)	*p*
Operation time (min)	223.9 (±77.8)	232.6 (±90.7)	0.368
Emergent operation	15 (16.1)	161 (8.5)	0.011
MIS	73 (78.5)	1481 (78.2)	0.945
Diversion	16 (17.2)	349 (18.4)	0.775
Duration of POD (days)	12.1 (±7.9)	13.4 (±11.9)	0.289
Complications	8 (8.6)	197 (10.4)	0.583
Complications ≥ 2	2 (2.2)	24 (1.3)	0.345
Mortality within 30 days	1 (1.1)	2.6 (1.4)	1.000
Use of CTx	58 (62.4)	860 (45.3)	0.002
5FU-LV/XELODA/UFT	16 (17.2)	356 (18.9)	
FOLFOX/XELOX	40 (43.0)	464 (24.6)	
FOLFIRI	1 (1.1)	20 (1.1)	
others	1 (1.1)	20 (1.1)	
Discontinuation of CTx	5 (8.8)	172 (20.0)	0.037
Multidrug regimen	42 (45.2)	512 (27.0)	<0.001

Data are presented as the number of patients (%) or mean (±standard deviation) unless otherwise stated. n, number; MIS, minimal invasive surgery; POD, postoperative days; CTx, Chemotherapy; 5FU-LV, Fluorouacil-leucovorin; UFT, Tegafur/Uracil; FOLFOX, Oxaliplatin + Fluorouacil + leucovorin; XELOX, Xeloda + Oxaliplatin; FOLFIRI, Irinotecan + Fluorouacil + leucovorin;.

**Table 3:** Pathologic outcome according to age.

	Young Adult Group (n = 93)	Older Group (n = 1899)	*p*
Histologic type			0.010
Well/moderate	82 (88.2)	1794 (94.6)	
Poorly/undifferentiated	11 (11.8)	103 (5.4)	
LVI	42 (45.2)	737 (38.8)	0.220
PNI	25 (26.9)	354 (18.7)	0.047
n of harvested LN	21.9 (±11.9)	23.2 (±15.1)	0.400
T			0.233
T0	4 (4.3)	82 (4.3)	
T1	10 (10.8)	311 (16.4)	
T2	12 (12.9)	236 (12.5)	
T3	55 (59.1)	1060 (55.9)	
T4	12 (12.9)	206 (10.9)	
N			0.670
N0	59 (63.4)	1218 (64.2)	
N1	20 (21.5)	441 (23.2)	
N2/N3	14 (15.1)	237 (12.5)	
TNM stage ^1^			0.785
0	5 (5.4)	83 (4.4)	
I	19 (20.4)	476 (25.1)	
II	36 (38.7)	665 (35.0)	
III	33 (35.5)	675 (35.5)	

Data are presented as the number of patients (%) or mean (±standard deviation) unless otherwise stated. n, number; LVI, Lymphovascular invasion; PNI, Perineural invasion; LN lymph node. ^1^ The tumor stage was defined according to the 8th edition of the American Joint Committee on Cancer TNM staging system.

**Table 4:** Univariate and multivariate analysis of overall survival.

Variable	Univariate		Multivariate	
	HR (95% CI)	*p*	HR (95% CI)	*p*
Age < 45	0.339 (0.106–1.083)	0.058	0.440 (0.139–1.392)	0.162
Men	1.042 (0.760–1.428)	0.800	0.954 (0.702–1.296)	0.763
ASA ≥ 3	1.799 (1.309–2.471)	<0.001	1.749 (1.272–2.404)	0.001
Obstruction	2.003 (1.380–2.906)	<0.001	1.818 (1.260–2.623)	0.001
Rectal cancer	1.221 (0.884–1.686)	0.225	1.435 (1.045–1.972)	0.026
T4	1.993 (1.319–3.010)	0.001	1.737 (1.140–2.646)	0.010
Presence of LN (+)	2.275 (1.660–3.117)	<0.001	2.727 (1.870–3.979)	<0.001
PNI	1.322 (0.911–1.920)	0.141	0.927 (0.626–1.371)	0.702
LVI	2.814 (1.593–2.993)	<0.001	1.717 (1.204–2.451)	0.003
Poorly/undifferentiation	1.932 (1.125–3.318)	0.015	1.561 (0.927–2.626)	0.094
Chemotherapy	0.778 (0.567–1.068)	0.120	0.344 (0.241–0.490)	<0.001

HR, Hazard Ratio; CI, Confidence interval; ASA, American Society of Anesthesiologists; LN lymph node; PNI, Perineural invasion; LVI, Lymphovascular invasion.

**Table 5:** Univariate and multivariate analysis of recurrence-free survival.

Variable	Univariate		Multivariate	
	HR (95% CI)	*p*	HR (95% CI)	*p*
Age < 45	0.392 (0.195–0.785)	0.006	0.438 (0.225–0.853)	0.015
Men	1.083 (0.871–1.347)	0.474	1.046 (0.860–1.273)	0.650
ASA ≥ 3	1.695 (1.354–2.123)	<0.001	1.757 (1.434–2.153)	<0.001
Obstruction	2.133 (1.625–2.799)	<0.001	1.689 (1.340–2.131)	<0.001
Rectal cancer	1.558 (1.248–1.945)	<0.001	1.695 (1.387–2.071)	<0.001
T4	2.906 (2.161–3.908)	<0.001	1.920 (1.485–2.482)	<0.001
Presence of LN (+)	2.723 (2.184–3.396)	<0.001	2.111 (1.661–2.683)	<0.001
PNI	2.092 (1.629–2.687)	<0.001	1.297 (1.024–1.641)	0.031
LVI	2.386 (1.916–2.972)	<0.001	1.510 (1.204–1.894)	<0.001
Poorly/undifferentiation	1.892 (1.258–2.845)	0.002	1.320 (0.933–1.867)	0.117
Chemotherapy	1.567 (1.260–1.947)	<0.001	0.696 (0.555–0.873)	0.002

HR, Hazard Ratio; CI, Confidence interval; ASA, American Society of Anesthesiologists; LN lymph node; PNI, Perineural invasion; LVI, Lymphovascular invasion.

## Data Availability

The data underlying this article will be shared upon reasonable request to the corresponding author.
